# Comorbidities associated with genetic abnormalities in children with intellectual disability

**DOI:** 10.1038/s41598-021-86131-3

**Published:** 2021-03-22

**Authors:** Jia-Shing Chen, Wen-Hao Yu, Meng-Che Tsai, Pi-Lien Hung, Yi-Fang Tu

**Affiliations:** 1 School of Medicine for International Students, I-Shou University, Kaohsiung, 84001 Taiwan; 2 Department of Pediatrics, National Cheng Kung University Hospital, College of Medicine, National Cheng Kung University, 138, Sheng-Li Road, North Dist., Tainan, 70403 Taiwan; 3 Department of Pediatrics, Kaohsiung Chang Gung Memorial Hospital and Chang Gung University College of Medicine, Kaohsiung, 83301 Taiwan; 4 Institute of Clinical Medicine, College of Medicine, National Cheng Kung University, Tainan, 70101 Taiwan

**Keywords:** Genetics, Neurology

## Abstract

Intellectual disability (ID) has emerged as the commonest manifestation of underlying genomic abnormalities. Given that molecular genetic tests for diagnosis of ID usually require high costs and yield relatively low diagnostic rates, identification of additional phenotypes or comorbidities may increase the genetic diagnostic yield and are valuable clues for pediatricians in general practice. Here, we enrolled consecutively 61 children with unexplained moderate or severe ID and performed chromosomal microarray (CMA) and sequential whole-exome sequencing (WES) analysis on them. We identified 13 copy number variants in 12 probands and 24 variants in 25 probands, and the total diagnostic rate was 60.7%. The genetic abnormalities were commonly found in ID patients with movement disorder (100%) or with autistic spectrum disorder (ASD) (93.3%). Univariate analysis showed that ASD was the significant risk factor of genetic abnormality (P = 0.003; OR 14, 95% CI 1.7–115.4). At least 14 ID-ASD associated genes were identified, and the majority of ID-ASD associated genes (85.7%) were found to be expressed in the cerebellum based on database analysis. In conclusion, genetic testing on ID children, particularly in those with ASD is highly recommended. ID and ASD may share common cerebellar pathophysiology.

## Introduction

Intellectual disability (ID) is a disability of intellectual and adaptive functions^1^. Deficits in intellectual functions include reasoning, problem solving, planning, abstract thinking, judgment, academic learning, and learning from experiences. Deficits in adaptive function mean failure to meet developmental and sociocultural standards for personal independence and social responsibility^1^. It is an important medical issue in the child development. In early childhood, the diagnosis of ID is based on documented substantial developmental delays (DD), including motor, cognitive, and speech delays. The world-wide prevalence of DD/ID is 1.5–3%^2,3^.

Underlying causes of DD/ID are very diverse, including brain malformation, metabolic disorders, brain traumatic injury, vascular disorders, nervous system infection, genetic abnormalities or even environmental factors^2–4^. Along with advancement in biomolecular knowledge, we become aware that the genetic contribution to DD/ID is ever more significant than previously thought and thus DD/ID itself has emerged as the commonest phenotypic manifestation of underlying genomic abnormalities^5,6^. Knowing the genetic etiology for DD/ID may help confirm the diagnosis that requires specific treatment and may also relieve uncertainty, improve the understanding of prognosis, and anticipate and manage associated medical and behavioral problems. Moreover, genetic diagnosis facilitates counseling, cascade testing and pre-natal testing where appropriate^7^. Thus, genetic testing is crucial for DD/ID children with unexplained etiology.

With the rapid development of next generation sequencing (NGS) technology, all kinds of disease-causing mutations, such as SNVs (single nucleotide variants), Indel (small insertion/deletion) and CNVs (copy number variants), can be detected rapidly and efficiently^8^. Recent reports showed that the genetic diagnostic rate in severe DD/ID patients is only around 12–20.8% based on chromosomal microarray (CMA), and 16–45% based on whole-exome sequencing (WES)^9–11^. Thus nearly half of DD/ID children remain undiagnosed despite the available CMA and WES tests. Children with DD/ID are at an increasing risk for a variety of comorbidities, including growth failure, epilepsy, ASD, ADHD, psychiatric illness, sensory (vision and hearing) impairments, skeletal issues and endocrine/metabolic dysfunctions^12^. It is therefore hypothesized that children with DD/ID are likely to have underlying genetic causes if they are also presenting additional phenotypes/comorbidities. High costs and relatively low diagnostic rates often result from advanced genetic testing; therefore, additional phenotypes or comorbidities which are associated with genetic abnormalities can increase the genetic diagnostic yield in affected DD/ID patients are valuable clues for pediatricians in general practice.

In our study, we used a sequential diagnostic scheme combining CMA and WES to conclude the genetic diagnosis in a group of moderate to severe DD/ID patients with unexplained etiology, and the risk phenotypes or comorbidities for genetic abnormalities were analyzed.

## Results

### Demographics of the study population

The present study recruited a total of 61 ID/DD patients with 37 (60.7%) males. The age at the time of enrollment ranged from 3 to 18 years (median, 6 years). The spectrum of additional phenotypes or comorbidities was listed at Table 1. About 77% of the enrolled patients displayed failure to thrive or short stature, 52.5% with microcephaly, 26.2% with facial dysmorphism, 36.1% with epilepsy, 34.4% with hypotonia and 24.6% with autism. Congenital abnormalities of internal organs involving heart, kidney or gastrointestinal tract accounted for 8–10% of the enrolled patients (Table 1).

**Table 1:** Demographic data, associated phenotypes and genetic abnormality in study population.

	Total population N = 61 No./%	CNV abnormality^a^ No./%^c^	Nucleotide variation^b^ No./%^c^	Gene abnormality No./%^c^
Gender (male)	37/60.7	9/24.3	16/43.2	25/67.6
**Associated comorbidities/phenotypes**				
Microcephaly	32/52.5	6/18.8	12/37.5	18/56.3
Failure to thrive	48/78.7	9/18.8	22/45.8	31/64.6
Short stature	48/77.0	8/16.7	19/39.6	27/56.3
Facial dysmorphism	16/26.2	1/6.3	8/50.0	9/56.3
Epilepsy	22/36.1	5/22.7	7/31.8	12/54.5
Movement disorder	5/8.2	2/40.0	3/60.0	5/100
Spasticity	14/23.0	3/21.4	6/42.9	9/64.3
Hypotonia	21/34.4	5/23.8	9/42.9	14/66.7
Autistic spectrum disorder	15/24.6	3/20.0	11/73.3	14/93.3
ADHD	4/6.6	1/25.0	2/50.0	3/75.0
Hearing impairment	6/9.8	0/0.0	4/66.7	4/66.7
**Organ involvement**				
Congenital heart disease	6/9.8	0/0.0	2/33.3	2/33.3
Congenital kidney malformation	5/8.2	0/0.0	3/60.0	3/60.0
Congenital GI malformation	5/8.2	0/0.0	2/40.0	2/40.0

*ADHD* attention-deficit hyperactivity disorder, *CNV* copy-number variation.

^a^CNV abnormality detected by chromosomal microarray.

^b^nucleotide variation detected by whole-exome sequencing.

^c^Denominator is the number of each items (in the second column of each row).

### Causative genetic variants in DD/ID patients

In 12 out of all 61 probands, CMA identified clinically significant 13 CNVs that were consistent with their phenotypes (Supplementary Table 1). The genetic diagnostic rate based on CMA was 19.7%. The other 49 patients with negative CMA results then underwent WES analysis. We further identified 24 variants in 25 probands (Supplementary Table 2). The 24 variants found in 18 genes included 8 pathogenic and 2 uncertain variants reported in ClinVAr, and 14 novel variants. Of the 14 novel variants, six truncating variants were classified as pathogenic, and 8 missense variants were either classified as pathogenic (n = 3) or likely pathogenic variants (n = 5) according to ACMG/AMP guidelines^13^. The overall genetic diagnostic rate using CMA and WES analysis was 60.7%.

### Risk factors of genetic abnormalities in DD/ID patients

Among the individuals having associated phenotypes and comorbidities, genetic abnormalities were most likely to be found in DD/ID patients with movement disorder (100%) or with ASD (93.3%; Table 1). Further risk analysis showed that there was no predicted risk associated with CNVs, but ASD was a factor highly associated with nucleotide variation (*P* = 0.002; OR 18.1, 95% CI 2.1–155.5) and overall genetic abnormalities (*P* = 0.003; OR 14, 95% CI 1.7–115.4; Table 2). In contrast, microcephaly, short stature, facial dysmorphism as well as movement disorder were not significantly associated with genetic abnormalities.

**Table 2:** Risk to genetic abnormality by CMA and/or WES.

	CNV abnormality (N = 12) No./%	*P* value	Nucleotide variation (N = 25) No./%	OR	P value	Gene abnormality (N = 37) No./%	OR	P value
Gender (male)	9/75.0	0.33	16/64	–	0.39	25/67.6	–	0.19
**Associated comorbidities/phenotypes**								
Microcephaly	6/50.0	1.00	12/48	–	0.57	18/48.6	–	0.60
Failure to thrive	9/75.0	0.71	22/88	–	0.17	31/83.8	–	0.34
Short stature	8/66.7	0.45	19/76	–	0.73	27/73.0	–	0.53
Facial dysmorphism	1/8.3	0.16	8/32	–	1.00	9/24.3	–	0.77
Epilepsy	5/41.7	0.74	7/28	–	0.37	12/32.4	–	0.59
Movement disorder	2/16.7	0.25	3/12	–	0.24	5/13.5	–	0.15
Spasticity	3/25.0	1.00	6/24	–	1.00	9/24.3	–	1.00
Hypotonia	5/41.7	0.74	9/36	–	0.76	14/37.8	–	0.59
Autistic spectrum disorder	3/25.0	1.00	11/44	18.1 (2.1–155.5)	0.02	14/37.8	14 (1.7–115.4)	0.003
ADHD	1/8.3	1.00	2/8	–	1.00	3/8.1	–	1.00
Hearing impairment	0/0.0	0.59	4/16	–	0.67	4/10.8	–	1.00
**Organ involvement**								
Congenital heart disease	0/0.0	0.59	2/8	–	0.42	2/5.4	–	0.20
Congenital kidney malformation	0/0.0	0.57	3/12	–	1.00	3/8.1	–	1.00
Congenital GI malformation	0/0.0	0.57	2/8	–	0.67	2/5.4	–	0.37

*ADHD* attention-deficit hyperactivity disorder, *CNV* copy-number variation, *OR (95% CI)* Odds ratio (95% confidence interval), *WES* whole-exome sequencing.

### ASD-associated genes in DD/ID patients

Of all 61 study patients, 14 out of 15 patients who had ASD were found to have genetic abnormalities including 3 CNVs and 11 nucleotide variations (Table 3). The regions of the 3 CNVs are located in chromosome Xp22.31, 15q11.2–13.1 and 17p11.2, and harbored genes associated with neurological diseases including GABRB3, UBE3A, MAGEL2, RAI1, ALDH3A2, ATPAF2 according to the database of Online Mendelian Inheritance in Man (OMIM). The 11 nucleotide variations involve genes including KDM5C, BRAF, KIF1A, IQSEC2, KMT2A, MECP2, SON, and DYRK1A. Among these genes, the most common gene harboring abnormalities was KDM5C that had been found in 3 patients, followed by KMT2A found in 2 patients. The tissue expression and biological functions of these ASD-associated genes in DD/ID patients were listed in Table 4. All of these 14 ASD-associated genes found in our DD/ID patients could be expressed in the central nerve system based on the GTEx database. Interestingly, we found that the majority of these genes (12/14) expressed in the cerebellum and a few are expressed in the frontal cortex and hypothalamus. The biological functions of these genes are involved in the transcriptional regulation, neurotransmission, cell proliferation, metabolism and protein stability, and all were essential for cellular homeostabsis in the central nervous system.

**Table 3:** Nucleotide variation/CNV abnormality list in 14 patients with autistic spectrum disorder.

Case	Nucleotide variation/chromosome abnormality	Gene
11	X:53228169 G>C, c.2233C>G	KDM5C
12	7:140453134 T>G, c.1801A>C	BRAF
14	X:53228169 G>C, c.2233C>G	KDM5C
16	2:241727608 G>A, c.223C>T	KIF1A
20	arr Xp22.31(6,560,639–7,029,767) × 2/0.469 Mb	HDHD1
22	arr 15q11.2q13.1(22,772,351–28,527,124) × 1/5.755 Mb	GABRB3, UBE3A, MAGEL2
23	arr 17p11.2 (16,790,077–19,868,384) × 3/3.078 Mb	*TNFRSF13B, FLCN, RAI1, ALDH3A2, ATPAF2, MYO15A, B9D1, AKAP10
33	X:53285127 G/−, c.854delC	IQSEC2
40	X:53224158 CT/−, c.3392_3393delAG	KDM5C
45	11:118365075 −/A, c.5250_5251insA	KMT2A
47	X:153296495 C>T, c.820C>T	MECP2
52	11:118353169 C/−, c.4048delC	KMT2A
60	21:34927288 TTAG/−, c.5753_5756delTTAG	SON
61	21:38865422 A>G, c.1028A>G	DYRK1A

*CNV* copy-number variation.

*Disease genes on OMIM.

**Table 4:** Tissue expression pattern and biological function in ASD-DD/ID associated genes.

Gene	Expression pattern (GETx)	Brain region with top two expression (median TPM)	Biological function (Uniprot)
KDM5C	Multiple tissues/whole brain	Cerebellum/cerebellar hemisphere	Histone demethylase regulates chromatin acetylation
BRAF	Multiple tissues/whole brain	Cerebellar hemisphere/cerebellum	Transduction of mitogenic signals, may play a role in postsynaptic responses of hippocampal neurons
KIF1A	Brain-specific/whole brain	Cortex/frontal cortex	Vesicle-mediated anterograde axonal transport
GABRB3	Brain-specific/whole brain	Frontal cortex/cerebellar hemisphere	Subunit of GABA inhibitory receptor in the brain
UBE3A	Multiple tissues/whole brain	Cerebellar hemisphere/cerebellum	E3 ubiquitin-protein ligase involves protein ubiquitination
MAGEL2	Multiple tissues/whole brain	Hypothalamus/pituitary	Enhances ubiquitin ligase activity
RAI1	Multiple tissues/whole brain	Cerebellum/cerebellar hemisphere	Transcriptional regulator of the circadian clock components, may be involved in neuronal differentiation
ATPAF2	Multiple tissues/whole brain	Cerebellar hemisphere/cerebellum	Assembly of the F1 component of the mitochondrial ATP synthase
ALDH3A2	Multiple tissues/whole brain	Cerebellar hemisphere/cerebellum	Catalyzes the oxidation of aliphatic aldehydes to fatty acids
IQSEC2	Brain-specific/whole brain	Cerebellum/cerebellar hemisphere	A guanine nucleotide exchange factor for the ARF GTP-binding proteins
KMT2A	Multiple tissues/whole brain	Cerebellum/cerebellar hemisphere	Histone methyltransferase that plays an essential role in early development and hematopoiesis
MECP2	Multiple tissues/whole brain	Cerebellar hemisphere/cerebellum	Chromosomal protein that binds to methylated DNA
SON	Multiple tissues/whole brain	Cerebellum/cerebellar hemisphere	mRNA splicing cofactor, essential for brain development, neuronal migration and metabolism
DYRK1A	Multiple tissues/whole brain	Cerebellar hemisphere/cerebellum	Regulate nuclear functions of cell proliferation

*GABA* γ-Aminobutyric acid, *GETx* Genotype-tissue expression protal (www.getxportal.org), *TPM* transcripts per kilobase million, *UniProt* database (www.uniprot.org).

## Discussion

Our research approach, which involved phenotyping, CMA and WES on the samples obtained from 61 consecutively enrolled probands with DD/ID with unexplained etiology, provided a diagnostic yield of 60.7%. Our diagnostic rate exceeds that of most published studies^9–11^. Totally, 13 CNVs (including 3 novel CNVs) and 24 nucleotide variations (including 14 novel variants) were revealed. We have provided information on variants that were possibly pathogenic in these genes based on available genetic evidence according to ACMG guidelines. Among these patients, we found overall genetic abnormalities were most likely to be found in the DD/ID patients with movement disorder or with ASD. Further univariate analysis showed ASD was a significant risk factor/comorbidity linked to genetic abnormalities in DD/ID patients with unexplained etiology and the genetic diagnostic rate was up to 93.3% in DD/ID patients with ASD. The biological functions of these ASD-DD/ID associated genes involved variable cellular functions in the central nerve system and the expression of these genes was mainly in the cerebellum.

Establishing a diagnosis of DD/ID children is critical for accurate health surveillance and further care planning for the affected individuals, especially for those with moderate-to-severe impairment. However, the etiology of DD/ID is very extensive and diverse^2–4^. Thus, to identify an etiology in DD/ID children is a challenge to clinicians, particularly in those who failed to obtain a diagnosis despite using the suggested stepwise systemic clinical evaluations^10,12,14^. The rapid development of NGS technology makes molecular genetic tests available in clinical practice, but its cost may not be affordable for most families. Children with DD/ID are at an increased risk for a variety of comorbidities, including growth failure, epilepsy, autistic spectrum disorder, attention deficits and hyperactivity disorder, psychiatric illness, sensory (vision and hearing) impairments, skeletal issues and endocrine/metabolic dysfunctions^12^. From these comorbidities, our study found that ASD was a risk factor/comorbidity associated with genetic abnormalities in DD/ID patients with unexplained etiology and the genetic diagnostic rate was up to 93.3% in DD/ID patients with ASD. Thus, genetic survey was deemed critical and should be highly encouraged in moderate to severe DD/ID children who also had the comorbidity of ASD.

Among the DD/ID children with ASD, we found multiple genetic variants in at least 11 ASD-DD/ID associated genes. The abnormalities of KDM5C and KMT2A genes were found in more than two patients. Interestingly, both genes are chromatin regulators and abundantly expressed in the brain^15,16^. KDM5C encodes a histone demethylase and KMT2A encodes a histone methyltransgerases^15,16^. KDM5C knockout mice as well as KMT2A knockout mice both exhibited abnormal social behavior including aggression, impaired learning and memory^15,16^. Chromatin stability and flexibility rely on dynamic regulations and are crucial for neuronal circuitries, synaptic plasticity and the development of the nervous system^17^. Dysregulation of chromatin regulation caused cognitive deficits and autistic behaviors^17^. In a most recent study, Satterstrom et al. sequenced 35,584 samples including 11,986 with ASD and identified 102 ASD-associated genes^18^. Among these 102 ASD-associated genes, 49 genes were associated with DD/ID and had more disruptive de novo variants than ASD-associated genes without DD/ID. Three ASD-DD/ID associated genes in our study (GABRB3, RAI1, and DYRK1A) were also revealed in Satterstrom et al. study^18^. The GABAAR β3 subunit (encoded by GABRB3) is important for type A γ-Aminobutyric acid (GABA_A_) receptor assembly, emerges at embryonic stage in the whole brain and reaches its strongest expression at the perinatal stage^19^. RAI1 encodes a transcriptional factor implicated in embryonic neurodevelopment, neuronal differentiation, cell growth and cell cycle regulation^20^. DYRK1A involves in neurogenesis, neuronal differentiation and dendrite formation during brain development^21,22^. Other ASD-DD/ID associated genes noted in our study also expressed in the brain and involved in the neurophysiology. Thus, cognitive deficits (DD/ID) and ASD are likely to be biochemically and molecularly related, and share common neuropathophysiology.

Based on the GTEx database, we found that the majority of ASD-DD/ID associated genes in our study are highly expressed in the cerebellum, and two in the frontal cortex. This finding indicates the significant role of cerebellum in the neuropathophysiology of DD/ID and ASD. In addition to neocortex, evidence obtained from clinical and neuroimaging studies has shown that cerebellum are involved in a series of cognitive functions^23–25^. Functional Magnetic Resonance Imaging studies showed that cognitive tasks of language, visual, spatial, executive and working memory can trigger cerebellar activation^26^. Lesions confined to the cerebellum result in cerebellar cognitive affective syndrome, the hallmark features of which include clinically relevant deficits in executive function, visual spatial performance, linguistic processing and dysregulation of affect. The affective dysregulation of autism spectrum disorder was manifested in deficits in emotional control, attentional control and social skills^27^. Moreover, hypoplasia or volume decreases in the cerebellar vermis was often identified in ASD children, and a correlation was found between these changes and the severity of behavioral and cognitive deficits, which include impairment social interaction, communication and increased repetitive behaviors^28^. Our findings were also supported by several computational studies using aggregated gene expression patterns to demonstrate that ASD susceptibility genes showed high coexpression in two distinct regions during brain development: the frontal/somatomotor neocortex and the cerebellar cortex^29,30^.

The relatively high diagnostic yield that we reported here may stem from the restrict inclusion criterion of moderate to severe DD/ID. Moreover, small case numbers in our cohort may lead to type I error and the wide 95% CI of the data in our subgroup analysis (Tables 1 and 2) impose a degree of uncertainty. Further studies recruiting a larger number of DD/ID patients will be necessary to validate our findings. Studies to validate or rule out causality of the candidate novel variants found in our study are ongoing. In conclusion, a sequential diagnostic scheme combining CMA and WES is useful to confirm the genetic diagnosis in a group of moderate to severe DD/ID patients with unexplained etiology. ASD was a risk factor/comorbidity associated with genetic abnormalities and increased the genetic diagnostic yield. Moreover, the ASD-DD/ID associated genes are highly expressed in the cerebellum, suggesting a role of cerebellum in the neuropathophysiology of DD/ID and ASD.

## Methods

### Study population

We enrolled consecutive 61 patients who aged below 18 years old and was diagnosed as moderate or severe DD/ID with unexplained etiology at our hospital, a tertiary referral medical center in Southern Taiwan, from Feb 2018 to Dec 2019. Moderate or severe DD/ID was defined by a performance score at least two standard deviations below the mean for an appropriate tests, including the Bayley Scales of Infant Development (BSID-III), or Wechsler Preschool and Primary Scale of Intelligence (WPPSI-IV), etc.^2,3^. All medical records and laboratory results, especially MR neuroimages and metabolic disorder surveys, were reviewed. Each enrolled patient was assessed in our pediatric department by a pediatric neurologist for detailed neurological examination and a pediatric geneticist for identification of facial dysmorphism or other phenotypes. Unexplained DD/ID was diagnosed when there was no any defined etiology of DD/ID after detailed clinical evaluation and available laboratory/imaging investigations. DD/ID patients with any possible known etiology were excluded. This study was approved by the ethics committee at National Cheng Kung University Hospital, and the parents of all patients in this study provide written informed consent. All the methods were carried out in accordance with the relevant guidelines and regulations.

### Covariates/comorbidities/phenotypes

Short stature refers to a body height which is more than two standard deviations below the mean for children of that same-sex and chronologic age, while failure to thrive refers to a weight for age that falls below the 5th percentile on multiple occasions or weight deceleration that crosses two major percentile lines on a growth chart^31,32^. Microcephaly is defined as a head circumference more than two standard deviations below the mean for gender and age^33^. When autistic trait or attention-deficit hyperactivity disorder (ADHD) was suspected during the assessments by psychologists, additional tests such as Continuous Performance Tests, Standard Version of the Childhood Autism Rating Scale—Second Edition or Social Communication Questionnaire were performed^34,35^. Diagnosis of autistic spectrum disorder (ASD) or ADHD was further confirmed by certificated pediatric psychiatrists based on the diagnostic criteria of the Diagnostic and Statistical Manual of Mental Disorders, fifth edition (DSM-V)^36^.

### CMA and WES analysis and validation

**Figure 1:**
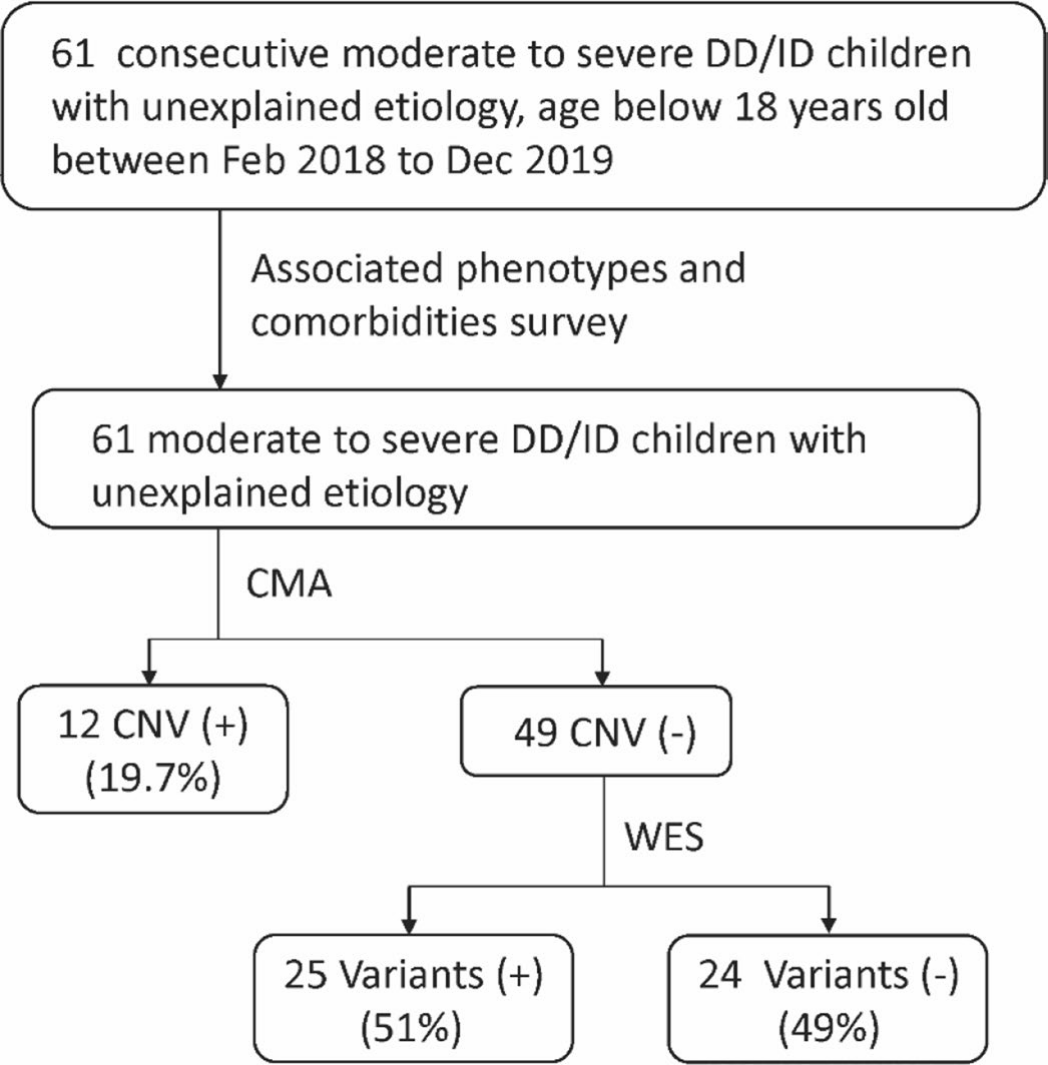
Flowchart of genetic survey. The developmental delay (DD)/intellectual disability (ID) children with unexplained etiology were tested with chromosomal microarray (CMA) and sequential whole-exome sequencing (WES). *CNV* copy-number variation.

Genomic DNA was isolated from blood of all study patients and their parents with the use of a FavorPrep Nucleic Acid Extraction kit and was stored at − 20 °C. All patients underwent CMA analysis first. WES was further tested to detect nucleotide variants in those who had negative results on CMA (Fig. 1).

For CMA analysis, all samples were screened using the CytoOneArray chromosomal DNA microarray (Phalanx Biotech group Inc., Taiwan, ROC). The resolution of this microarray is nearby 30 Kb, especially focused on disease-related genes. The experimental processes were executed by Phalanx service laboratory which was certificated by ISO17025CNVs calling were obtained by using circular binary segmentation (CBS) algorithm which are conducted by the function of MATLAB version R2009a (Math Works Inc.). Following the ACMG guidance for copy-number variation (CNV), the detected CNVs were interpreted according to whether the CNVs were pathogenic or benign in the scientific literature, general genome databases, integrated databases, and curated databases, including UCSC Genome Browser website, OMIM, ClinGen Dosage Sensitivity Map, dbVar database, Database of Genomic Variants, International Standards for Cytogenomic Arrays Consortium Database and DECIPHER^37,38^.

For WES, exomes were enriched with the use of a SeqCap EZ MedExome Target Enrichment Kit (Roche Sequencing, USA). The enriched DNA samples were sequenced by 2 × 150 paired-end sequencing using the nextseq500 high output sequencing system (Illumina, USA) to produce raw data. The paired-end sequence reads obtained from Illumina NovaSeq 6000 platform were filtered and trimmed to retrieve high-quality reads. After processing alignment, quality check, variant calling, annotation, and prioritization, we classified the candidate variants as pathogenic, likely pathogenic, uncertain significance, and benign, according to the guidelines from the ACMG. Candidate pathogenic or likely pathogenic variants were scrutinized for genotype to phenotype analysis and manually reviewed by using ClinVar and OMIM database^13,39^. The final diagnosis or causative variants was confirmed using Sanger sequencing.

Tissue expression of disease associated genes was assessed using publicly available data on the GTEx database (www.gtexportal.org), and the biological function of disease associated genes was obtained from the UniProt database (www.uniprot.org).

### Statistics

In order to identify the predictive factors of the genetic diagnostic yield, we compared differences in yield rate between the presence and absence of certain demographic and clinical factors using the Fisher exact test. Further, potential predictors with *P* < 0.05 in the univariate analysis or clinically significant variables were included in a multivariate logistic regression model. Adjusted odds ratios (ORs) and associated 95% confidence intervals (CIs) were computed. All analyses were performed on SPSS (version 20.0; SPSS Institute, Chicago, IL, USA).

## Supplementary Information

Supplementary Tables.
