# Supplementary material for: Comorbidities associated with genetic abnormalities in children with intellectual disability
Source: Sci Rep. 2021 Mar 22;11:6563. doi: 10.1038/s41598-021-86131-3 (PMC7985145; doi:10.1038/s41598-021-86131-3)
Supplement: Supplementary file 1 — Supplementary Tables. [file 41598_2021_86131_MOESM1_ESM.docx]

**Comorbidities associated with genetic abnormalities in children with intellectual disability**

Jia-Shing Chen, PhD^1^, Wen-Hao Yu, MD^2^, Meng-Che Tsai, MD, PhD^2^, Pi-Lien Hung, MD, PhD^3^, Yi-Fang Tu, MD, PhD ^2,4, *^

^1^ School of Medicine for International Students, I-Shou University, Kaohsiung 84001, Taiwan

^2^ Department of Pediatrics, National Cheng Kung University Hospital, College of Medicine, National Cheng Kung University, Tainan 70403, Taiwan

^3^ Department of Pediatrics, Kaohsiung Chang Gung Memorial Hospital and Chang Gung University College of Medicine, Kaohsiung 83301, Taiwan

^4^ Institute of Clinical Medicine, College of Medicine, National Cheng Kung University, Tainan 70101, Taiwan

**Supplementary Table 1. List of ID/DD associated CNV from CMA**

| Case | CNV from CMA | Syndromic diagnosis |
| --- | --- | --- |
| 3 | arr 5q12.1-q12.2(60,817,239-62,964,539)x1/2.147Mb | Chromosome 5q12 deletion syndrome |
| 9 | arr 14q32.33(106,761,195-107,135,147)x1/0.374 Mb | VOU, likely pathogenic ^Maurin et al. 2006^ |
| 17 | arr 8p23.3p23.1(215,647-11,247,757)x1/11.032Mb  arr 8q24.13q24.3(126,008,251-144,053,532) x3/18.045Mb | Recombinant 8 syndrome |
| 18 | arr 15q13.3(32,024,772-32,444,010)x3/0.419Mb | Chromosome 15q13.3 microduplication syndrome |
| 20 | arr Xp22.31(6,560,639-7,029,767)x2/0.469Mb | Xp22.31 duplication |
| 22 | arr 15q11.2q13.1(22,772,351-28,527,124)x1/5.755Mb | Angelman syndrome |
| 23 | arr 17p11.2(16,790,077-19,868,384)x3/3.078Mb | Potocki-Lupski syndrome |
| 27 | arr 2p16.1p15(60,984,162-61,765,384)x3/0.781Mb | microduplication syndrome involving 2p16.1-p15 |
| 29 | arr 21q22.13(37,834,467-38,105,036)x3/0.271Mb | VOU, likely pathogenic ^Pelleri et al. 2016^ |
| 38 | arr 16p13.11(15,131,575-16,288,874)x3 / 1.157-Mb | 16p13.11 microduplication syndrome |
| 39 | arr 9p24.2(3454536-3479862)x1 (25Kbp) | VOU, likely pathogenic ^Chen et al. 2018^ |
| 44 | arr Xq25(122,997,689-123,506,416)x2/0.509Mb | Xq25 duplication syndrome |

CMA: chromosomal microarray

CNV: copy number varinant

VOU: variants of uncertain significance

**Reference**

Chen B, Niu J, Kreuzer J, Zheng B, et al. Auto-fatty acylation of transcription factor RFX3 regulates ciliogenesis. Proc Natl Acad Sci USA. 2008;115: E8403-E8412.

Maurin ML, Brisset S, Le Lorc'h M, et al. Terminal 14q32.33 deletion: genotype-phenotype correlation. Am J Med Genet A. 2006; 140:2324-2329.

Pelleri MC, Cicchini E, Locatelli C, et al. Systematic reanalysis of partial trisomy 21 cases with or without Down syndrome suggests a small region on 21q22.13 as critical to the phenotype. Hum Mol Genet. 2016;25:2525-2538.

**Supplementary Table 2. List of ID/DD associated nucleotide variation from WES**

| Case | Gender | OMIM | | Genomic position | Zygosity | NM number | cDNA level | Protein level | Classification | Significance |
| --- | --- | --- | --- | --- | --- | --- | --- | --- | --- | --- |
|  |  | Gene | Inheritance |  |  |  |  |  |  |  |
| 1 | M | GNAO1 | AD | 16:56370747 A>C | Hetero | NM_020988.2 | c.698A>C | p.Gln233Pro | missense | pathogenic |
| 2 | M | SLC16A2 | XL | X:73641840 TCCTCTAC/- | Hemi | NM_006517.4 | c.371_378delTCTACTCC | p.Leu124Hisfs*40 | LOF | pathogenic |
| 6 | F | mTOR | AD | 1:11217312 A>G | Hetero | NM_004958.3 | c.4366T>C | p.Trp1456Arg | missense | pathogenic |
| 7 | M | MED12 | XLR | X:70343543 T>C | Hemi | NM_005120.2 | c.1717T>C | p.Phe573Leu | missense | likely pathogenic |
| 11 | M | KDM5C | XLR | X:53228169 G>C | Hemi | NM_004187.3 | c.2233C>G | p.Gln745Glu | missense | likely pathogenic |
| 12 | M | BRAF | AD | 7:140453134 T>G | Hetero | NM_004333.4 | c.1801A>C | p.Lys601Gln | missense | pathogenic |
| 14 | M | KDM5C | XLR | X:53228169 G>C | Hemi | NM_004187.3 | c.2233C>G | p.Gln745Glu | missense | likely pathogenic |
| 16 | M | KIF1A | AD/AR | 2:241727608 G>A | Hetero | NM_004321.7 | c.223C>T | p.Arg75Trp | missense | uncertain |
| 19 | F | KIF1A | AD/AR | 2:241727535 G>A | Hetero | NM_004321.7 | c.296C>T | p.Thr99Met | missense | pathogenic |
| 24 | M | KCNQ2 | AD | 20:62070997 G>A | Hetero | NM_172107.3 | c.881C>T | p.Ala294Val | missense | pathogenic |
| 28 | F | COQ4 | AR | 9:131088128 G>A | Homo | NM_016035.5 | c.370G>A | p.Gly124Ser | missense | uncertain |
| 33 | M | IQSEC2 | XLD | X:53285127 G/- | Hemi | NM_001111125.2 | c.854delC | p.Pro285Leufs*21 | LOF | pathogenic |
| 40 | M | KDM5C | XLR | X:53224158 CT/- | Hemi | NM_004187.3 | c.3392_3393delAG | p.Glu1131Alafs*72 | LOF | Pathogenic |
| 43 | M | ANKRD11 | AD | 16:89347302 -/A | Hetero | NM_013275.5 | c.5648dupT | p.Ser1884Leufs*66 | LOF | Pathogenic |
| 45 | F | KMT2A | AD | 11:118365075 -/A | Hetero | NM_001197104.1 | c.5250_5251insA | p.Ala1753Serfs*32 | LOF | Pathogenic |
| 47 | F | MECP2 | XLD/XLR | X:153296495 C>T | Hetero | NM_001110792.2 | c.820C>T | p.Gln274Ter | LOF | pathogenic |
| 48 | F | GFM1 | AR | 3:158409182 C>T | Homo | NM_001308164.1 | c.2239C>T | p.Gln747Ter | LOF | pathogenic |
| 49 | F | GNAO1 | AD | 16:56370758 G>A | Hetero | NM_020988.2 | c.709G>A | p.Glu237Lys | missence | pathogenic |
| 52 | F | KMT2A | AD | 11:118353169 C/- | Hetero | NM_001197104.1 | c.4048delC | p.Arg1350Alafs*6 | LOF | pathogenic |
| 54 | M | TRIO | AD | 5:14485247 G>A | Hetero | NM_007118.3 | c.6727G>A | p.Gly2243Ser | missence | likely Pathogenic |
| 55 | F | BRAF | AD | 7:140477853G>C | Hetero | NM_004333.4 | c.1455G>C | p.Leu485Phe | missence | pathogenic |
| 56 | M | BRAF | AD | 7:140449165T>A | Hetero | NM_004333.4 | c.1914T>A | p.Asp638Glu | missence | pathogenic |
| 59 | M | BRPF1 | AD | 3:9781045 T>C | Hetero | NM_001003694.1 | c.962T>C | p.Leu321Pro | missence | likely Pathogenic |
| 60 | F | SON | AD | 21:34927288 TTAG/- | Hetero | NM_032195.2 | c.5753_5756delTTAG | p.Val1918Glufs*87 | LOF | pathogenic |
| 61 | M | DYRK1A | AD | 21:38865395A>G | Hetero | NM_001396.4 | c.1028A>G | p.Asp343Gly | missence | likely Pathogenic |

OMIM: Online Mendelian Inheritance in Man; M: male; F: female; AD: autosomal dominant; AR: autosomal recessive; XL: X-linked; XLD: X-linked dominant; XLR: X-linked recessive; Hetero: heterozygous, Homo: homozygous, Hemi: hemizygous, LOF: loss of function: WES: whole-exome sequencing
